# Short- and medium-term effects of biodegradable microplastics (PLA and PHB) on earthworm development and reproduction

**DOI:** 10.1007/s10646-025-02868-x

**Published:** 2025-03-17

**Authors:** David Gutiérrez-Rial, Aarón Lagoa, Iria Villar, Pilar Feijoo, Fuencisla Mariño, Josefina Garrido, Benedicto Soto, Salustiano Mato

**Affiliations:** 1 Department of Ecology and Animal Biology, Faculty of Biology, University of Vigo, Vigo, Spain; 2 Department of Plant Biology and Soil Science, Faculty of Biology, University of Vigo, Vigo, Spain

**Keywords:** Ecotoxicity, Growth, Fecundity, Biopolymer, Emerging contaminants, Eisenia andrei

## Abstract

Microplastics derived from biobased and biodegradable materials will increase their presence in soils as their use becomes more widespread. Research into their effects on soil fauna will help to ensure a better understanding of their environmental impacts. The aim of this work was to study the effects on the development of the earthworm *Eisenia andrei* (ingestion capacity, survival, growth, cocoon, and hatchling production), earthworm lysosomal stability through the neutral red retention time (NRTT), and substrate enzymatic activity of dehydrogenase (DHA) and fluorescein diacetate-hydrolysing activity (FDA) in the presence of polylactic acid (PLA), polyhydroxybutyrate (PHB) and polyethylene (PE) microplastics in laboratory tests. Three different tests were designed, one feeding test of 4 days, and two medium-term tests with 49 and 112 days. The 4-day test and the 49-day growth test were carried out using OECD artificial soil, while in the 112-day growth test, vermicompost was used as the substrate. PLA and PHB particle ingestion was demonstrated. No concentration or polymer-dependent lysosomal damage or effects on earthworm growth were observed. However, reproductive effects, such as a decrease in cocoon production and the number of juveniles, were reported upon exposure to PE and PLA during medium-term assays. These findings indicated that the toxicity of PLA bioplastic exposure is comparable to that of conventional plastic PE concerning the negative effects on the reproductive efficiency of the detritivorous earthworm *E. andrei*.

## Introduction

Because of ubiquitous microplastic (MP) pollution, alternative materials such as bioplastics are becoming increasingly popular. Bioplastics, according to European Bioplastics (2023), are either biobased, biodegradable, or those materials that may have both properties. Thus, taking it into account, materials such as biodegradable plastics from fossil-based feedstock, non-biodegradable but bio-based forms of traditional plastics, and bio-based and biodegradable materials from renewable feedstocks are classified as bioplastics. But it is of great importance to clarify the distinction between the term bioplastic in general and biodegradable, as not all bioplastics are prone to the biodegradation process (Rosenboom et al. 2022).

Bioplastics production currently accounts for around 1% of the global plastics market, but it is expected to grow in coming years. Consequently, a greater amount of bioplastic waste will need to be managed, otherwise, it will end up in natural environments (Chah et al. 2022). These plastic materials must meet standards to be labelled as biodegradable but the conditions under which these tests are conducted do not always take place in nature. Over the last decade, research on bioplastics increased exponentially (Haider et al. 2019) and the knowledge about the behaviour of bioplastics in the environment, both in laboratory (Sedničková et al. 2018; Bandopadhyay et al. 2020) and field studies (Rudnik and Briassoulis, 2011; Touchaleaume et al. 2016; Kalita et al. 2021), has increased considerably. Recent reviews claim that most plastics, including many reported as biodegradable, are more prone to disintegration than degradation and it is important to remark that biodegradability only depends on the chemical accessibility of the material to microorganisms, while disintegrability can be affected by factors such as shape or size of the sample (de Souza Machado et al. 2018; Gadaleta et al. 2024).

Most studies on the effects of MPs from bioplastics have shown significant effects, but only at concentrations in soil of at least 1% w/w. However, these doses are much higher than those found in natural soils (Rosenboom et al. 2022; Jiang et al. 2024). Soil-dwelling invertebrates, especially soil-feeding invertebrates, are highly exposed to substances present in the environment, including MPs of different natures. One of the most widely used groups in soil ecotoxicology studies is the oligochaetes. Their ecological characteristics mean that they have the capacity to increase the bioavailability of many toxic substances to other organisms through ingestion, digestion, assimilation in the gut, and excretion of compounds associated with different pollutants (Singh, 2018). Earthworms can transport and fragment the plastic particles present in the medium through the processes of excavation (Huerta Lwanga et al. 2017; Zhang et al. 2018), but they have also been found to have the ability to ingest fragments of these polymers (Huerta Lwanga et al. 2016; Lahive et al. 2022). Several studies have highlighted the harmful effects of this exposure on different earthworm species (Huerta Lwanga et al. 2016; Rodriguez-Seijo et al. 2017; Chen et al. 2023). However, earthworms have predominantly been used in ecotoxicity studies involving petroleum-derived plastics. There has been less research conducted on the use of earthworms in ecotoxicity studies involving bio-based and biodegradable polymers. While Liwarska-Bizukojc et al. (2022) and Rodríguez et al. (2023) found no harmful effects on earthworms with polylactic acid (PLA)-based bioplastics, Huerta-Lwanga et al. (2021) suggested that the presence of PLA residues in compost could affect *L. terrestris* mortality rate or affect survival. Considering the limited understanding of the effects of bioplastics on soil fauna and the absence of ecotoxicological studies on the presence of polyhydroxybutyrate (PHB) MPs, it is crucial to expand our knowledge of the potential impact of bioplastics on soil, especially with the increasing presence of bioplastics in the terrestrial ecosystem.

The objectives of the feeding test and medium-term laboratory studies with the earthworm *Eisenia andrei* Bouché, 1972 and PLA, PHB, and polyethylene (PE) MPs were: 1) To determine if there is any difference in the type of MPs (PLA, PHB, PE) and the size of MPs that earthworms are able to ingest, in the feeding test (4 days); 2) To assess the ecotoxicological effect of MPs on *E. andrei* over a medium-term experiment (49 days), testing different MPs doses to determine their impact on earthworm growth and reproduction; 3) To evaluate the ecotoxicological effect of a single concentration of MPs in a medium-term experiment (112 days) in an organic-rich substrate, testing earthworm growth, reproduction, and substrate enzymatic activity.

## Material and methods

### Earthworms

Specimens of the earthworm *E. andrei* were collected from a laboratory culture, which was fed with horse manure. The collected earthworms were rinsed, weighted, and subsequently placed in petri dishes with moistened filter paper for 24 h to empty their gut content, one individual per petri dish. The specimens were weighed again with empty guts to be selected for each of the different tests.

### Test biopolymers

Poly-β-hydroxybutyrate (PHB) pellets (product name ENMAT Y3000P, Ningbo Tianan Biologic Material Co., Ltd) and polylactic acid (PLA) pellets (product name PLI 005, NaturePlast) were reduced to particles smaller than 1000 µm using a cryogenic grinder CryoMill (Retsch, Haan, Germany). The maximum length of a total of 100 particles of each micronized polymer was measured under the stereomicroscope Leica S9D by using Leica Application Suite X (LAS X, version 3.7.2, Leica Microsystems, Wetzlar, Germany) to determine the average particle size: PHB 0.446 mm ± 0.218, PLA 0.418 mm ± 0.293. Polyethylene powder (PE) low density 500 µm (A10239) was supplied by Thermo Scientific Chemicals.

### Feeding test in petri dishes

Petri dishes (90 × 14 mm) were filled with 35 g of standardised soil and 5 g of horse manure, previously frozen for at least 48 h to avoid the presence of cocoons (fresh weight). Standard soil was prepared according to the OECD (OECD, 2004) with a mixture (based on dry weight) of 10% sphagnum peat, 20% kaolin clay, 0.3–1% CaCO_3_, 70% air-dried quartz and the pH was adjusted to 6 by adding calcium carbonate. One individual of clitellated *E. andrei*, with a mean weight of 0.269 ± 0.050 g, was placed on each plate under exposure to six different MPs concentrations (A: 0.03125%, B: 0.0625%, C: 0.125%, D: 0.250%, E: 0.50%, F: 1.00%, dry weight). Five replicates for each biopolymer (PHB and PLA) and five controls without MPs were tested. The treatments were incubated in darkness at 20 °C ± 2 and 65% ± 5 of moisture for 4 days. After the incubation time, earthworms were removed from the plates, rinsed, weighted, depurated for gut emptying during 24 h, and reweighted. After the incubation time the survival rate for each treatment was measured and the earthworm casts were collected in an Eppendorf tube and mixed with 1 ml of distilled water using the vortex to afterwards analyse the presence of MPs in the casts. The number of MPs was counted, and the size of the particles was measured under the stereomicroscope Leica S9D using Leica Application Suite X (LAS X, version 3.7.2, Leica Microsystems, Wetzlar, Germany).

### Medium-term artificial soil test

To test ecotoxicological effects on *E. andrei*, 500 ml culture systems were filled with 150 g of standardised soil, as exposed in the survival test, and 50 g of previously frozen horse manure. Four pre-clitellate individuals of *E. andrei*, with an average weight of 0.209 ± 0.039 g, were exposed to three different MPS concentrations (A: 0.0625%, B: 0.125%, C: 0.250%, dry weight) of three different polymers PHB, PLA and PE. Concentrations were selected according to the work of Rodriguez-Seijo et al. (2017). Each treatment was prepared in triplicate and three controls without MPs were included. The treatments were incubated at 20 ± 2 °C and 65 ± 5% moisture with a photoperiod of 8 h light /16 h dark for 49 days (7 weeks) and no additional feed was provided during the experiment to maintain the concentration of MPs in the culture systems throughout the incubation time. The survival /mortality rate, the weight of the earthworms (all four earthworms with the full intestinal tract), and cocoon production were measured weekly. To determine cocoon hatching success, cocoons were placed in individual wells of microplates and hatched was controlled according with Porto et al. (2012). At the end of the experiment, earthworms were removed, rinsed, weighed, depurated for gut emptying during 24 h, and reweighted. Two earthworms per replicate of each treatment and concentration were tested for lysosomal membrane stability with neutral red retention time assay (NRTT).

### Medium-term vermicompost test

To test the ecotoxicological effects over time on *E. andrei*, a total of 80 culture systems of 500 ml were filled with 200 g of commercial vermicompost from chicken and horse manure and 50 g of horse manure previously frozen to feed the earthworms. This experiment was developed using vermicompost as substrate due to its higher nutrient content and higher moisture retention capacity than artificial soil. Four pre-clitellated specimens of *E. andrei* were placed on each culture system (average weight 0.217 ± 0.056 g). Twenty replicates of each treatment of MPs (PLA, PHB, and PE) were spiked at 0.125% (w:w), plus twenty control replicates were prepared (without MPs). The treatments were incubated at 20 ± 2 °C and 65 ± 5% moisture with a photoperiod of 8 h light /16 h dark for 112 days (16 weeks) and, as in the previous experiment, earthworms were not fed during the incubation period. Every 28 days, 5 replicates of each treatment were removed and analysed. At each sampling time, survival rate, earthworm development (weight variation), cocoon and hatchling production, and cocoon hatching success were determined. Additionally, each substrate was sampled for physico-chemical and enzymatic analysis.

### Biochemical analysis

#### Cellular damage on earthworms

Lysosomal membrane stability was analysed by applying the neutral red retention time technique (NRRT) (Lowe et al. 1995). Briefly, a volume of 20 μL of coelomic fluid containing coelomocytes was collected by 4.5 V of electric shock. Celomic fluid was mixed with an equal volume of temperature adjusted (15 °C) earthworm physiological Ringer solution. The solution was placed onto a microscope slide and the cells were allowed to adhere to the slide for 30 s prior to the application of an equal volume of the neutral red working solution (80 µg ml^−1^) and a cover slip. The slides were kept in a humidity chamber when not under observation. Each slide was observed under a microscope at 2-min intervals until 50% of the cells showed leakage of the lysosomal membrane, or for 60 min if no damage was detected, as done by Booth et al. (2001). The interval was recorded as the neutral-red retention time.

#### Enzymatic activity measured on substrate

Moisture and organic matter contents of soil samples were calculated gravimetrically after drying at 105 °C until constant weight and combustion at 550 °C for 4 h, respectively. Dehydrogenase activity (DHA) on vermicompost samples was determined by the reduction of 2,3,5-triphenyltetrazolium chloride (TTC) to triphenyl formazan (TPF) according to Alef and Nannipieri (1995). Briefly, 1 g of substrate was incubated with 1 mL of 1.5% TTC for 24 h at 30 °C. TPF was measured spectrophotometrically at 550 nm. DHA activity was expressed as μg TPF per g dry weight of soil. Fluorescein diacetate-hydrolyzing activity (FDA) on vermicompost samples was analysed as described by Alef and Nannipieri (1995). Briefly, 1 g of substrate was incubated with fluorescein diacetate (10 µg ml^−1^) in a shaker for 3 h at 24 °C. Fluorescein liberated was measured spectrophotometrically at 490 nm. FDA was expressed as μg fluorescein per g dry weight per hour.

### Data analysis

All statistical tests were performed using R software (R Core Team, 2023). First, the Shapiro-Wilk test was applied to investigate the distribution of the different variables. After testing for normal distribution, to investigate whether the earthworms’ weight varied between polymers and concentrations in the feeding test (4-days), an analysis of variance (ANOVA) factorial model was conducted. Differences among polymer size in the feeding test were determined with Wilcoxon-Mann-Whitney test.

Earthworms’ weight in the medium-term artificial soil test (49-days) was analysed using linear mixed-effects models (LMMs) with the lme4 package. The type of polymer and the concentration were fixed factors and the repeated measurement throughout time was treated as a random effect to address the non-independence of the measurements within every individual over time. Model comparison and selection were based on the Akaike Information Criterion (AIC). The data for reproductive efficiency (total number of cocoons, total number of hatchlings, and hatchling per cocoon) were each subjected to a one-way analysis of variance (ANOVA) using the Tukey post hoc test for contrast of the differences among treatments. The nonparametric Kruskal-Wallis test and the multiple comparison test (kruskalmc) between treatments for non-normal variables were used.

The earthworm weight data (total weight and weight gained) from the medium-term vermicompost test (112-days) were each subjected to two-way ANOVA with time and polymer as factors. Negative binomial regression was used to test the effects of polymer and time in hatchling and cocoon abundance, while a linear mixed model was applied to test the effect of polymer and time on the enzymatic activities of the substrates.

All statistical tests were evaluated at the 95% confidence level and values are given as the mean ± standard deviations.

## Results

### Feeding test

**Fig. 1:**
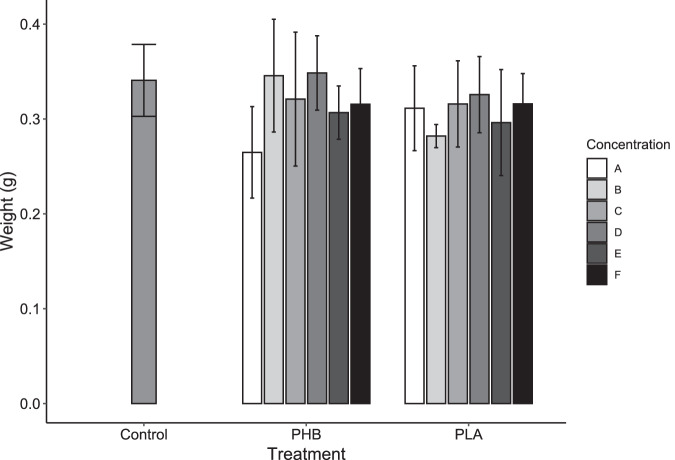
Mean weight of E. andrei with the different polymers (PLA, PHB) and concentrations (A: 0.03125%, B: 0.0625%, C: 0.125%, D: 0.250%, E: 0.50%, F: 1.00%) and Control without MPs after the 4-day test. Error bars represent standard deviations (*N* = 5). No significant differences were found among groups

All individuals survived the test regardless of the concentration and type of polymer. There were not significant differences between treatments at the beginning of the test. At the end of the experiment, earthworm´s weight was significantly greater than the initial after 4 days (*p* < 0.05), but no significant effects of polymer type or concentration on final weight and weight gain per earthworm were found (Fig. 1).

**Fig. 2:**
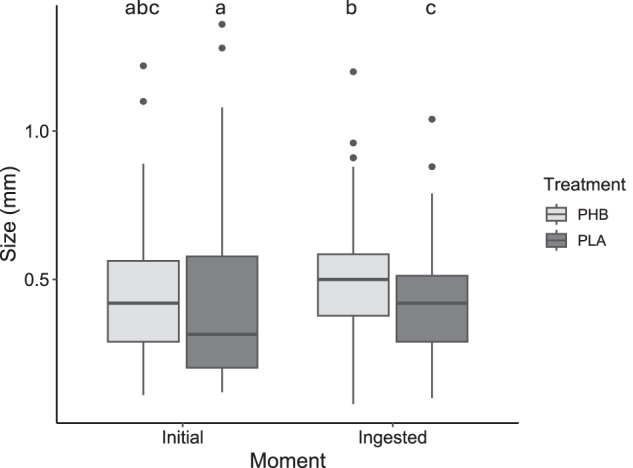
Particle size distribution at the beginning of the experiment and after the experiment by analysing the MPs content in the casts of E. andrei after the -test in the PHB and PLA treatments. Different letters show statistically significant differences (*p* < 0.05)

As shown in the Fig. 2, no differences in particle size were observed between PLA and PHB at the beginning of the experiment (*p* > 0.05) and MPs were detected in all replicates of the different treatments through the observation of casts. In terms of ingested particle size, statistically significant differences were found between PLA and PHB after 4 days (*p* < 0.05), with a smaller average size in the case of PLA (0.431 ± 0.174 mm) compared to that found in PHB (0.489 ± 0.209 mm) (Fig. 2). In addition, differences were found between PLA particles at the beginning and after 4 days, with a larger average particle size in the casts (0.431 ± 0.174 mm) than at the start of the experiment (0.412 ± 0.227) (*p* < 0.05). All individuals of *E. andrei* ingested MPs during the 4 days of the experiment and no effect on growth was noted.

### Medium-term artificial soil test

**Fig. 3:**
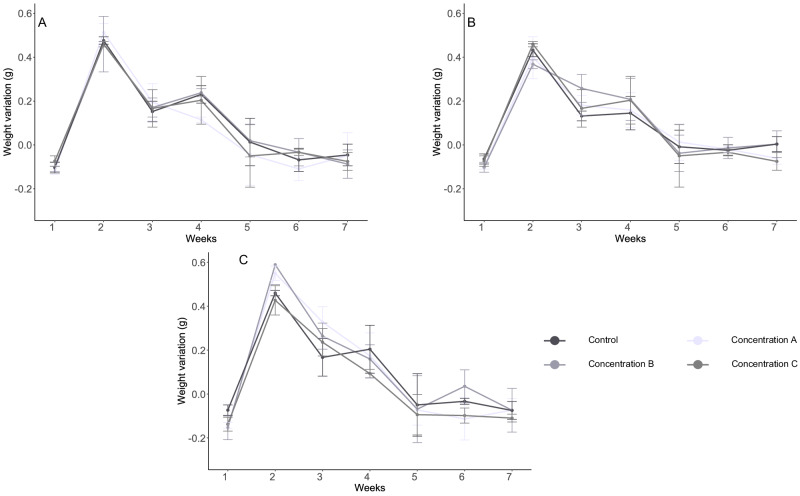
Weight variation experienced by E. andrei individuals after exposure to different concentrations of different treatments compared to control: **A**) PHB. **B**) PLA and **C**) PE. Error bars represent standard deviations (*N* = 3)

The linear model that included polymer and time but excluded the three-way interaction (time, polymer and concentration) was the most appropriate model for explaining the variations in the weight variable. Earthworm weight was significantly affected by both time and polymer (*p* < 0.001). Notably, changes in earthworm weight were primarily attributed to the effect of time since all sampling times showed significant differences (*p* < 0.001). As can be seen in Fig. 3, earthworms began to lose weight in all treatments after the fourth week. Also, the treatment with PLA polymer exhibited a statistically significant effect on earthworm weight (*p* < 0.05), while the other polymers had no significant effects. The PLA treatment showed lower earthworm growth throughout the whole process in comparison with PHB, PE and control treatments. These results indicated that other factors than polymer concentration played a significant role in determining earthworm weight, with the time and PLA polymer being the primary drivers of the observed differences.

**Fig. 4:**
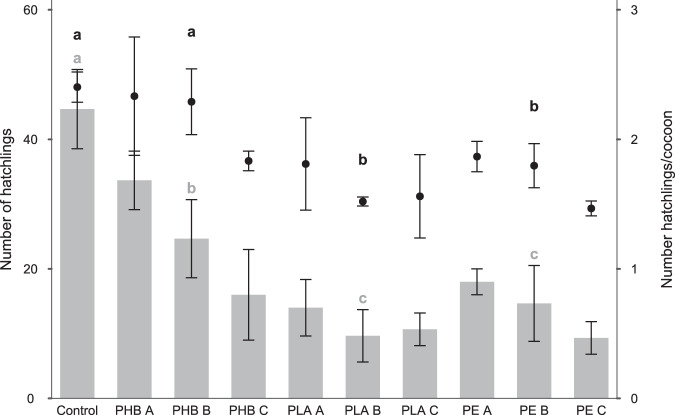
Bars represent the total number of hatchlings found in each treatment and dots represent the number of hatchlings per cocoon of E. andrei counted under the exposure to the different polymers (PLA, PHB, PE, and C: Control without MPs) and concentrations (A: 0.062 5%, B: 0.125%, C: 0.250%) of the medium-term artificial test. Error bars represent standard deviations (*N* = 3). Lowercase letters mean significant differences between treatments (grey for number of hatchlings, black for number of hatchlings per cocoon)

No significant differences were found in the total number of cocoons between the treatments (*p* > 0.05), with a mean value of 23.9 ± 5.1. However, statistically significant differences were detected in the number of hatched cocoons (*p* < 0.05), the number of total hatchlings (*p* < 0.001), and the ratio of hatchlings per cocoon (*p* < 0.01). While the mean hatching percentage in the control treatment was 67.7 ± 16.7, the mean percentage in PHB, PE and PLA were 44.2 ± 8.3, 33 ± 3.8 and 35.1 ± 1.7, respectively. Therefore, exposure to MPs resulted in reduced reproductive efficiency compared to the Control treatment (Fig. 4). Also, the presence of PHB microplastics at low concentrations (A and B) could exert a lesser effect on the decrease in reproductive efficiency when compared to the addition of PLA and PE MPs. Finally, as indicated by the NRTT assay, no cellular damage was detected in the lysosomes of adult earthworms from any of the treatments following the completed tests.

### Medium-term vermicompost test

Earthworms’ weight was not affected by the interaction time and polymer (*p* > 0.05). Notably, changes in earthworm weight were primarily attributed to the effect of time since all sampling times showed significant differences (*p* < 0.001). A decrease in earthworm growth was observed over time in all treatments (Table 1). The highest earthworm weight gain was measured at 28 days in all treatments and the longer the incubation time the lower the earthworm growth without statistically significant differences attributable to the polymer type.

**Table 1:** Changes in total weight gain, number of hatchlings and number of cocoons of E. andrei with the different polymers (PLA, PHB, PE and Control without MPs) of medium-term vermicompost test over time

	Time (Days)	Gain weight (g)	N° Cocoons	N° Hatchlings
Control	28	0.87 ± 0.16	12.0 ± 9.7	–
	56	0.58 ± 0.19	13.8 ± 12.1	23.0 ± 12.5
	84	0.34 ± 0.24	2.8 ± 1.5	63.8 ± 27.3
	112	0.23 ± 0.15	–	47.2 ± 23.1
PHB	28	0.89 ± 0.14	11.0 ± 5.1	–
	56	0.91 ± 0.52	10.4 ± 8.6	20.0 ± 10.2
	84	0.38 ± 0.16	–	47.2 ± 14.0
	112	0.24 ± 0.19	–	39.0 ± 6.9
PLA	28	0.95 ± 0.05	8.6 ± 6.7	–
	56	0.65 ± 0.09	7.8 ± 3.3	16.4 ± 10.0
	84	0.39 ± 0.06	–	49.4 ± 28.8
	112	0.19 ± 0.07	–	48.4 ± 29.4
PE	28	0.94 ± 0.15	6.4 ± 4.5	–
	56	0.60 ± 0.23	11.4 ± 6.6	19.8 ± 7.9
	84	0.47 ± 0.22	–	17.8 ± 10.7
	112	0.27 ± 0.13	–	28 ± 21.1

Data are mean ± standard deviation (*N* = 5)

The negative binomial regression model that includes polymer and time interaction, was the most appropriate for explaining the variations in hatchling and cocoon abundance (Table 1). Significant interactions were found in cocoon abundance by polymer and time (*p* < 0.001). The number of cocoons was significantly higher in the Control treatment than in the PE and PLA treatments (*p* < 0.05). The number of cocoons found decreased in all treatments over time, with a significant effect at 84 days (*p* < 0.001). No significant interactions were found in hatchling abundance by time but there was a significant effect of PE treatment (*p* < 0.01) with a lower number of hatchlings compared to the Control.

**Fig. 5:**
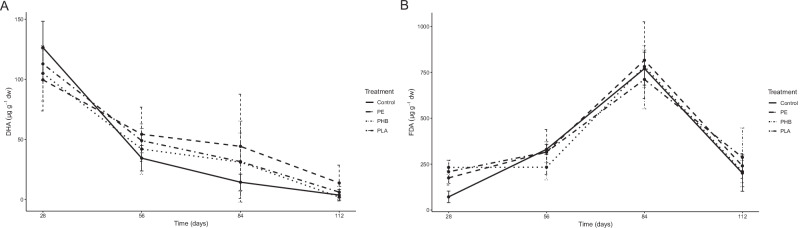
Changes in **A**) dehydrogenase activity (DHA) and **B**) Fluorescein diacetate-hydrolyzing activity (FDA) over time in the substrate with the different polymers (PLA, PHB, PE and Control without MPs) of medium-term vermicompost test. Data are mean ± standard deviations (*N* = 5)

In the case of DHA (Fig. 5A), there was a strong decrease over time in all treatments and a significant joint effect of polymer and time was found (*p* < 0.001). In particular, the PE treatment showed higher activity than the Control (*p* < 0.05) with a greater effect on 56 and 84 days. All treatments showed a similar trend in FDA with an increase up to 84 days and then a drastic decrease up to 112 days (Fig. 5B). A significant joint effect (*p* < 0.001) between polymer type and time was found, indicating that the combination of these factors significantly influences FDA activity over time. Significant differences (*p* < 0.05) in this activity were observed for the PHB and PLA treatments compared to the Control, but no clear trend over time was detected.

## Discussion

### MPs in casts

Previous studies have shown that the ecotoxicity tests with model organisms such as *E. andrei* are greatly dependent on the level of exposure to the pollutants (Okoffo et al. 2021). This is conditioned by the exposure time (Huerta Lwanga et al. 2017; Rodriguez-Seijo et al. 2017) but also by the physical and chemical properties such as particle size (Rosenkranz et al. 2009; Cole et al. 2013; Besseling et al. 2015). Additionally, other factors such as the shape of the MPs may affect their bioavailability to interact with other pollutants in the soil or to be ingested by the organisms that inhabit it (Wright et al. 2013).

Plastics, whether biodegradable or not must be present in sufficient quantity or for a long enough period to be consumed by the organisms. In addition, if the particles have some property such as large size, they cannot be consumed by the target organism, so ingestion effects cannot be evaluated. However, toxicity could arise from leaching contaminants such as plastic additives (Talsness et al. 2009) through earthworm tegument exposure apart from intestinal uptake. These factors are crucial to simulate realistic conditions and accurately assess the impacts that MPs may have on detritivorous fauna in laboratory conditions. In this research, the average particle size of MPs was around 450 µm. *E. andrei* earthworms can ingest particles that pass through their mouth with the help of their prostomium and body contractions. Mechanical ingestion of MPs particles is not limited since the mouth size of most temperate earthworms is 3 mm (Rodriguez-Seijo et al. 2017), so the PHB and PLA particles can be ingested without any issues as was demonstrated by cast observation.

The particle size observed after ingestion (Fig. 2) indicated a possible selection of particles depending on the polymer type — a selection of smaller particles of PLA — or a polymer degradation during the passage through earthworms’ gut. Meng et al. (2023) pointed out that no size-dependent selection during ingestion of PLA were observed in a 4-day exposition of *Lumbricus terrestris* to MPs as only food source. However, Cui et al. (2022) exposed that earthworms can selectively ingest MPs, preferring those with smaller particle size and biodegradable compositions. Meng et al. (2023) also observed that PLA MPs could fragment and depolymerise in the earthworm gut. This agrees with Wang et al. (2022) who reported that PLA MPs were broken down into smaller fractions than PET after its passage through the gut of *E. fetida*. No information is available on the biodegradation of PHB in the presence of earthworms compared to PLA. Although, Al Hosni et al. (2019) showed that PLA degrades earlier than PHB under thermophilic conditions during composting, whereas Zhang and Thomas (2011) observed that raw PLA does not biodegrade at room temperature whereas raw PHB shows rapid biodegradation at room temperature during biodegradation test with compost. Although selection or degradation of PLA could not be determined, the ecotoxic effects of ingesting both MPs could be studied.

### Survival and development of earthworms

As shown in previous studies, the presence of MPs derived from biodegradable polymers at low concentrations does not cause any negative effects on earthworm survival or growth (Huerta-Lwanga et al. 2021; Holzinger et al. 2023) in short- to medium-term trials. These results support the findings of this study in which no statistically significant differences in mass loss and survival of *E. andrei* were found between treatments. Jager (2004) observed that even when manure is present a large part of the diet of *E. andrei* consists of soil particles when they are incubated on artificial substrates. In this study, the earthworms were fed at the start and left without additional feeding to avoid a significant decrease in the MPs concentration in the substrates during exposure. The earthworms experienced weight loss over time both with and without MPs, in the two medium-term treatments. The lack of continuous feeding or provision of additional food throughout the study led to the ingestion of previously digested food by the earthworms, so that the compounds pass through the intestinal tract more times, increasing the potential degradation and the potential toxicity of MPs. However, the low concentration or type of polymers used did not exert a greater toxic response in earthworms, including the conventional polymer PE. Huerta Lwanga et al. (2016) found that the growth rate of *L. rubellus* was negatively affected by high concentrations (>28% w/w) of smaller PE particles in leaf litter during a 60-day experiment. In contrast, Rodriguez-Seijo et al. (2017) observed no effects on the survival and body weight of *E. andrei* on 28 days of exposure to concentrations less than 1000 mg/kg of PE MPs although intestinal damage was detected. According to this, Yang et al. (2021) showed no body weight inhibition of *E. fetida* exposed to concentrations below 2% of PE MPs but changes in metabolic enzyme activities were found at exposure concentrations equal to or greater than 0.25%. In this regard, the NRRT did not indicate a toxic effect on lysosomal membrane integrity of earthworms due to the presence of different concentrations and polymers in the standardised soil. It is important to note that the MPs particle size used in this study was too large to permit direct cellular uptake (Riedl et al. 2022). Consequently, NRRT was employed to detect lysosomal damage in the event of significant particle disintegration and degradation or due to the presence of toxic additives in the MPs used. NRRT is considered a sensitive early biomarker to predict pollution stress in soil (Svendsen et al. 2004; Shi et al. 2007) and was used to measure earthworm toxicity response when studying the combined effect of MPs and organic pollutants (Boughattas et al. 2022; Shi et al. 2022). The results demonstrated the absence of lysosomal toxicity, indicating that neither the disintegration nor degradation of the particles nor the potential release of toxic additives were sufficient to cause discernible cellular damage under the conditions of the experiment.

Most studies detecting harmful effects on earthworms are generally conducted at much higher concentrations than those present in the natural environment and usually with oil-based polymers (Fuller and Gautam, 2016; Scheurer and Bigalke, 2018; Ding et al. 2021). In this study, the analysis of the casts showed that the presence of bioplastic particles with sizes below 500 microns represents in itself a potential threat to these organisms as they can be ingested as previously observed by Cui et al. (2022). Furthermore, the harmful effect of MPs derived from bio-based or biodegradable polymers could be worse if the tested polymers had incorporated additives that during degradation processes could be released into the environment or into the gut of earthworms (Sridharan et al. 2022). It has often been shown that additives can be more toxic to the fauna than MPs themselves, in fact Novo et al. (2018) found modified endocrine pathways in the male organs with altered gene expression of *E. fetida* after 28 days of exposure to BPA (a known endocrine disruptor).

### Reproductive effects

The results showed the ability of the *E. andrei* earthworms to reproduce in substrates with different types of polymers. The decrease in bioavailable nutrients accessible to earthworms with time, inhibited the growth and reproduction of *E. andrei* in both the short and medium-term tests. However, a different response was observed depending on the substrate and/or processing time. In the short-term standardised soil, negative effects on hatchling efficiency and number of hatchlings were detected mainly in PLA and PE. In the medium-term vermicompost, the negative impact on earthworm fertility is due to the presence of PE more than PLA. Several studies have reported that MPs may have an adverse effect on earthworms’ reproduction. For example, Ding et al. (2021) reported a significant reduction in *E. fetida* cocoons and juveniles with high doses of MPs, with a major effect of PE than biodegradable MPs. Yang et al. (2021) also showed negative effects on reproduction of *E. fetida* during a continuous exposure to 20 g/kg PE MPs for 60 days. Damage of reproductive organs of *E. andrei* was observed by Kwak and An. (2021). This previous work showed that spermatogenesis was inhibited and also detected abnormal growth in seminal vesicle tissues as a consequence of earthworms’ exposure to PE MPs during 21 days. Exposure to PE and PLA in the medium-term study carried out with artificial soil showed a similar effect on reproduction, which would indicate that both conventional and biodegradable polymers have a toxic effect on reproduction. Nevertheless, the number of hatchlings found in PE treatments was much lower than in the others, indicating greater effects as a consequence of the exposure to the conventional polymer rather than biodegradable polymers in the experiment with vermicompost. Zimmerman et al. (2020) showed that biodegradable materials available on the market are just as toxic as conventional plastics with regards to the chemicals they contain. The polymers used in this study were analytical grade powder PE and PLA and PHB micronized from commercial pellets. The concentration of additives in these polymers may be sufficiently low to not cause harm to cells or the development of earthworms.

### Microbial activity

Dehydrogenase activity (DHA) and fluorescein diacetate-hydrolyzing activity (FDA) are considered to represent the microbial oxidative capability and microbial hydrolytic capability during organic matter decomposition process, respectively (Huang et al. 2023) and are often used to characterize soil microbial activity. It was demonstrated that soil microbial communities can be affected by the presence of MPs with changes in microbial structure and composition (Yi et al. 2021). Therefore, the presence of MPs in soil can influence enzymatic activities (Sajjad et al. 2022). DHA is an intracellular enzyme, assumed to be very sensitive to the environmental stress that affects living soil microorganisms, so the limited nutrients over time could reduce microbial biomass and therefore this enzymatic activity (Wolinska and Stepniewsk, 2012). On the contrary, no sharp decline of FDA was observed. This enzymatic activity depends on the contribution of both extracellular and intracellular enzyme activities (Nannipieri et al. 2003), so the overall microbiological activity had a similar trend through time in all polymers. However, the presence of MPs could stimulate the microbial activity, assessed as FDA, at the initial of the experiment similar to the findings of (Liu et al. 2017). Similarly, the presence of PE MPs displayed greater DHA with a less significant decline in activity than the control. On the contrary, Shah et al. (2023) found that PE negatively affected DHA in soil after 40 days in the presence of soybean. Although altered soil properties resulting from microplastics are proposed as a possible reason for the changes in soil microbial activities, no solid evidence or robust linkages have been verified (Wang et al. 2020).

## Conclusions

The rise of innovative materials such as bioplastics as an alternative to conventional plastics requires the evaluation of their potential impacts on the environment and the fauna that sustains it. The results obtained in this study showed that exposure of *E. andrei* to PHB, PLA and PE MPs have no deleterious effects on survival and growth rate in short- and medium-term experiments. Nevertheless, the exposure to PLA and PE MPs caused a reduction of the reproductive fitness in the short-medium-term experiments, resulting in a lower number of cocoons and a lower hatchling rate compared to that found in the PHB and control treatments. However, it is important to note that the materials used in this study lacked additives in their composition, indicating that further research is needed to understand how the presence of such substances might affect these results

## Data Availability

Data will be available under request to the corresponding author.
